# Targeting mitochondria in bone and cartilage diseases: A narrative review

**DOI:** 10.1016/j.redox.2025.103667

**Published:** 2025-05-07

**Authors:** Daniel H. Mendelsohn, Nike Walter, Wing-Hoi Cheung, Ronald Man Yeung Wong, Rebecca Schönmehl, Lina Winter, Thaqif El Khassawna, Christian Heiss, Christoph Brochhausen, Markus Rupp

**Affiliations:** a Institute of Pathology, Medical Faculty Mannheim, Heidelberg University, Mannheim, Germany; b Department of Psychosomatic Medicine, University Medical Center Regensburg, Regensburg, Germany; c Department of Orthopedics and Traumatology, The Chinese University of Hong Kong, Hong Kong Special Administrative Region of China; d Experimental Trauma Surgery, Justus-Liebig-University Giessen, Germany; e Department of Trauma, Hand and Reconstructive Surgery, University Hospital Giessen, Germany; f Biruni University, Istanbul, Türkiye; g Friedrich-Baur-Institute, Department of Neurology, LMU Clinic Munich, Germany

**Keywords:** Mitochondria, Bone, Cartilage, Mitochondrial dynamics, Osteoarthritis, Osteoporosis, Osteomyelitis

## Abstract

Mitochondria are essential regulators of bone health, controlling cell differentiation, cellular energy production, immune function, osteogenesis, and osteoclast activity. Their dysfunction is linked to orthopedic disorders such as osteoporosis, osteoarthritis, and osteomyelitis, contributing to impaired bone homeostasis and increased fracture risk. While mitochondrial research has been more advanced in fields such as cardiology and neurology, emerging therapeutic strategies from these areas are beginning to show potential for translation into orthopedics. These include mitochondrial biogenesis stimulation, mitochondrial fission inhibition, antioxidant therapies, mitochondrial transplantation, and photobiomodulation, which have demonstrated success in enhancing tissue repair, reducing oxidative stress, and improving overall cellular function in non-orthopedic applications. The novel inhibitor of mitochondrial fission and accumulation of reactive oxygen species Mdivi-1 offers potential to improve clinical outcomes of bone diseases by alleviating cellular dysfunction and preventing bone loss. While these treatments are still in the developmental phase, they present innovative approaches to address mitochondrial dysfunction in orthopedic conditions, potentially transforming bone disease management and enhancing patient outcomes. This report explores research regarding the involvement of mitochondrial health in bone and joint function and discusses possible future treatment strategies targeting mitochondria in orthopedic conditions.

## Graphical abstract

**Figure:**
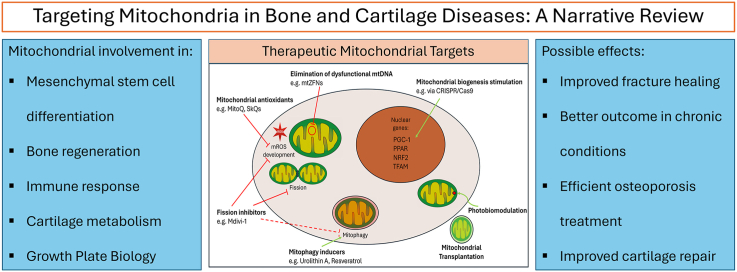

## 1 Introduction

Mitochondria, the cellular powerhouses, are integral to numerous physiological processes, including energy production, regulation of metabolic pathways, and apoptosis [1]. Their role in orthopedics has gained attention due to their impact on bone metabolism, repair mechanisms, and the pathophysiology of musculoskeletal disorders [[2], [3], [4]]. However, mitochondrial health has been explored to a smaller extent in orthopedics compared to other fields such as cardiology or neurology, where it has been extensively studied [5]. With the emergence of novel treatment strategies targeting mitochondria, it is crucial to deepen our understanding of mitochondrial involvement in orthopedic conditions. This narrative review examines the multifaceted roles of mitochondria in skeletal health, explores the potential therapeutic strategies targeting mitochondrial function, and discusses the implications for clinical practice in orthopedics.

**Fig. 1:**
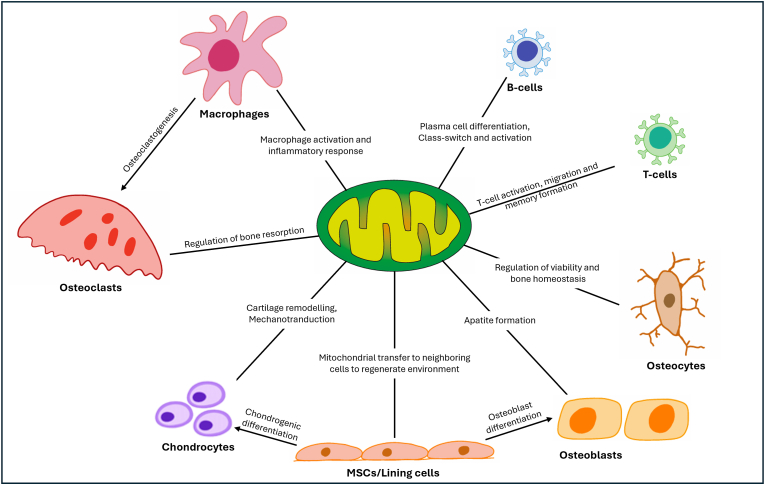
Schematic depiction of the involvement of mitochondria in cellular function in bone tissue. Lines radiating from central mitochondrion are labeled with the primary function that mitochondria are involved with. Arrows indicate differentiation processes in which mitochondria play a crucial role.

Mitochondria play a crucial role in bone health by regulating the balance between osteogenesis and osteoclast activity, cellular energy production through oxidative phosphorylation (OXPHOS), redox signaling via reactive oxygen species (ROS), apoptosis, and calcium homeostasis (Fig. 1) [6,7]. They also support nucleotide and amino acid synthesis, which is important for cell proliferation and extracellular matrix production [8,9]. Disruptions in mitochondrial function can impair bone remodeling and contribute to diseases such as osteoporosis and osteoarthritis [[10], [11], [12]].

It is important to note that in the orthopedic conditions discussed in this review, mitochondrial dysfunction generally occurs as a secondary phenomenon rather than as a primary defect. Unlike primary mitochondriopathies, such as mitochondrial DNA depletion syndromes, orthopedic diseases like osteoporosis, osteoarthritis, and osteomyelitis involve mitochondrial alterations secondary to factors such as hormonal deficiency, mechanical stress, chronic inflammation, or infection [13,14]. Recognizing this distinction is critical for interpreting the role of mitochondrial dysfunction within the broader context of disease pathogenesis.

Despite the growing recognition of mitochondrial involvement in orthopedic pathologies, the existing literature largely addresses these mechanisms in isolation or within specific cell types. This review seeks to integrate current insights on mitochondrial dynamics, quality control, and immunometabolism across a broad spectrum of bone and cartilage-related conditions. By highlighting emerging treatment strategies and cross-disciplinary links—particularly from cardiology, immunology, and stem cell biology—this review aims to provide a comprehensive and clinically relevant synthesis. In doing so, it underscores underexplored therapeutic potentials and positions mitochondria as central regulators of orthopedic disease and healing.

### 1.1 Mitochondria in osteocyte function

Osteocytes, the most abundant bone cells, play crucial roles in bone homeostasis and metabolism [15]. Recent research has revealed their complex energy metabolism, involving both glycolysis and OXPHOS [16]. Notably, osteocytes can transfer mitochondria to adjacent stressed cells via the endoplasmic reticulum, a process mediated by Mfn2, which helps restore metabolic function [17]. This mitochondrial transfer also plays a role in regulating angiogenesis in transcortical vessels [18]. Furthermore, oxidative metabolism and mitochondrial activity vary among cortical bone osteocytes, with cells near bone surfaces exhibiting higher mitochondrial content [19]. Arguably, these differences may in part result from the mechanosensory functions of osteocytes [20], as the mitochondrial network is mechanosensitive and acts as an integral part of the cell's mechanosensing apparatus [21]. Recent studies have shown that mitochondrial mechano-transduction involves dynamic changes in mitochondrial morphology and activity in response to cyclic mechanical loading [22]. These adaptations include mitochondrial elongation, altered membrane potential, and increased ATP production, enabling osteocytes to meet the high energy demands of mechanotransduction [23]. Mechanosensitive pathways such as AMPK and cytoskeletal-mitochondrial coupling appear to coordinate these responses, linking physical stimuli to mitochondrial bioenergetics [24]. The transcription factor NRF2 is also crucial for osteocyte gene expression and bone homeostasis, with its activity increasing during osteocytogenesis in response to elevated ROS levels, with mitochondria being the main source of cellular ROS [25]. Since osteocytes are the most abundant cells in bone tissue, comprising over 90 % of cells within the bone matrix or on bone surfaces [15], understanding the mitochondrial role in osteocyte function is crucial for developing treatments for bone disorders and improving outcomes in osseointegration of medical devices [26].

Bone cell metabolism is shaped by the interplay between glycolysis and mitochondrial OXPHOS modulated by local oxygen tension [16]. Osteocytes, residing in hypoxic environments deep within the bone matrix, rely more heavily on glycolysis, whereas osteoclasts and osteoblasts on vascularized surfaces predominantly utilize mitochondrial respiration [27,28]. Hypoxia-inducible factors (HIFs) serve as key mediators of metabolic adaptation, promoting glycolytic gene expression while maintaining mitochondrial function [29,30]. Additionally, signaling molecules like Wnts not only regulate skeletal development but also influence mitochondrial dynamics and ER-Golgi trafficking, underscoring the integrated role of the organellar connectome in bone cell biology [31].

### 1.2 Mitochondria in osteoblast function

Osteoblasts, derived from mesenchymal stem cells, depend on mitochondrial dynamics for their differentiation and matrix production [32]. Early differentiation is marked by mitochondrial biogenesis and fission via dynamin-related peptide 1 (DRP1) [33], while maturation shifts towards fusion (via optic atrophy 1/OPA1), enhancing ATP generation and function [34,35]. Interestingly, mature osteoblasts exhibit increased mitochondrial fragmentation and donut formation – meaning the formation of circular structures with central holes, which enhance mitochondrial secretion and promote osteogenesis in osteoprogenitors [36]. The SIRT3/SOD2 antioxidant pathway protects against mitochondrial stress during osteogenesis [37]. Advanced oxidation protein products (AOPPs) can induce apoptosis through mitochondrial ROS and membrane depolarization, which is mitigated by mitophagy—the selective degradation of damaged mitochondria by autophagy, allowing the cell to maintain mitochondrial health and function [38]. Furthermore, mitochondria are involved in apatite formation as they serve as calcium phosphate storage sites and are involved in transporting these minerals to the extracellular matrix (ECM) [39,40]. Targeting osteoblastic mitochondrial function may provide novel therapeutic strategies for osteoporosis and other chronic bone diseases [2].

Mitochondrial quality control—including fusion, fission, biogenesis, and mitophagy—plays a critical role in osteoblast differentiation and function [2,34,35]. Proper regulation ensures the removal of damaged mitochondria and supports cellular adaptation during matrix production. Sirtuins, particularly SIRT3 and SIRT1, have emerged as key mediators of mitochondrial integrity in osteoblasts by modulating ROS detoxification, metabolic reprogramming, and differentiation pathways [37]. Disruption of these processes can impair osteogenesis and contribute to bone fragility [41].

### 1.3 Mitochondria in osteoclast function

Osteoclasts, derived from hematopoietic stem cells, are responsible for bone resorption during remodeling [42]. Mitochondrial dysfunction can disrupt the balance between osteogenesis and osteoclast activity, contributing to the pathogenesis of osteoporosis [2]. Mitochondria play critical roles in supporting osteoclast differentiation, energy production, and survival [43]. During osteoclast formation, mitochondrial biogenesis increases to meet energy demands, while mitochondrial quality control mechanisms, including fusion, fission, and mitophagy ensure healthy function [44,45]. Autophagy-dependent mitochondrial function plays an important role for osteoclast differentiation and maturation, with autophagic activity positively correlating with osteoclast activity and survival [46]. The co-activator PGC1β plays a pivotal role by coordinating mitochondrial biogenesis and cytoskeletal organization needed for resorption. Impairments in these processes weaken osteoclast function and bone remodeling [47].

Mitochondrial ROS production in osteoclasts is involved in signaling pathways that regulate bone resorption [43]. Excessive mitochondrial ROS can lead to enhanced osteoclast activity, contributing to bone loss in diseases like osteoporosis and rheumatoid arthritis [48]. Mitochondrial quality control regulates osteoclast homeostasis [46]. Fusion and fission dynamics influence energy production and resorptive activity, while mitophagy ensures the turnover of dysfunctional mitochondria. OXPHOS remains a major ATP source during active bone resorption, and impaired OXPHOS has been linked to defective cytoskeletal organization and reduced bone degradation capacity [27]. These findings highlight the complex relationship between mitochondria and osteoclast function, emphasizing their importance in bone homeostasis and potential therapeutic targets for bone diseases.

### 1.4 Mitochondria in chondrocyte function

Mitochondria are essential for chondrocyte function and cartilage homeostasis [49]. They regulate matrix calcification and survival through calcium handling and ROS signaling. Excessive mitochondrial fission via the ERK1/2–DRP1 pathway is associated with cartilage degradation [50], whereas TGFβ3-driven fission supports growth and metabolism [51]. Growth plate chondrocytes exhibit specialized mitochondria with elevated calcium transport capacity, essential for matrix ossification [52]. Phosphate-induced apoptosis of hypertrophic chondrocytes occurs through a mitochondrial pathway, involving changes in membrane potential and cytochrome *c* release [53,54]. Mitochondrial dysfunction has been observed in osteoarthritic chondrocytes, leading to reduced activities of respiratory chain complexes, decreased ATP levels, and altered membrane potential [10,55]. These changes contribute to increased chondrocyte apoptosis and decreased collagen production [11]. Mechanical stress, a major risk factor for osteoarthritis, is capable of inducing mitochondrial alterations in chondrocytes [23,56]. Mitochondria also act as mechanotransducers, linking extracellular mechanical signals to intracellular responses [23]. Therapeutically, strategies like hyaluronic acid supplementation can protect chondrocytes from oxidative damage and restore mitochondrial DNA repair [57]. Targeting mitochondrial metabolism in chondrocytes holds promise for novel cartilage regeneration therapies [58].

### 1.5 Mitochondria in immune system function

Mitochondria play a central role in both innate and adaptive immunity by supporting energy demands, regulating ROS production, and controlling calcium signaling [59,60]. They regulate immune cell activation, differentiation, and survival [61]. In T cells and B cells, mitochondrial bioenergetics directly impact cytokine release, class switching, and long-term immunity [[62], [63], [64], [65], [66], [67]]. Mitochondrial dysfunction has been linked to immunodeficiencies and increased susceptibility to infections [60]. Recent findings have underscored the importance of mitochondrial metabolism in regulating immune responses that directly affect bone remodeling. In macrophages, the balance between pro-inflammatory M1 and anti-inflammatory M2 phenotypes is tightly linked to mitochondrial function and ROS production, influencing osteoclast activation and inflammatory bone loss [44,68,69]. Similarly, in T cells, mitochondrial bioenergetics control cytokine release and survival, which can modulate bone resorption in chronic infections [70]. These insights are especially relevant in osteomyelitis, where immune-driven inflammation and mitochondrial dysfunction synergize to disrupt bone regeneration [71]. Integrating mitochondrial immunometabolism into the study of bone health offers a valuable perspective for understanding and treating osteo-inflammatory conditions. Understanding the diverse roles of mitochondria in immune responses provides insights into immuno-metabolism and potential therapeutic targets for immune-related disorders [61,62]. However, recent literature doesn't address the immune system specific to bone tissue. It is vital to further assess this in future studies. The involvement of mitochondrial dysfunction in the pathogenesis of osteomyelitis, the infection of bone and bone marrow, has already been demonstrated [71].

## 2 Mitochondria and potential therapeutic strategies

**Fig. 2:**
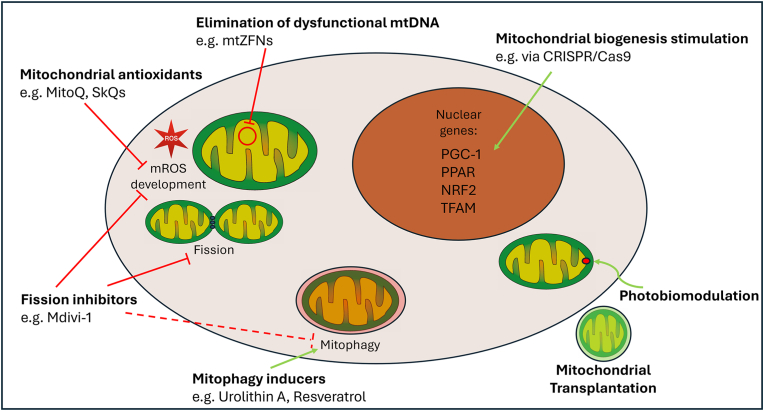
Schematic overview of the various treatment strategies targeting mitochondria. Green arrows indicate a stimulating effect. Red T-lines indicate an inhibitory effect. The dotted T-line from fission inhibitors to mitophagy indicates the contradictory results regarding this effect. While some articles propose that Mdivi-1 inhibits, others say it induces mitophagy.

**Fig. 3:**
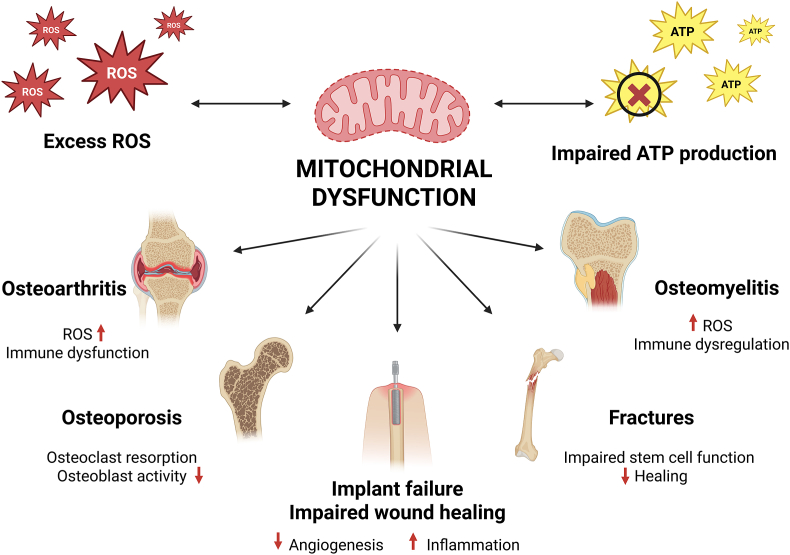
Overview of the impact of mitochondrial dysfunction in bone and cartilage diseases. Key features of mitochondrial impairment—such as excess reactive oxygen species (ROS), disrupted fission/fusion dynamics, and impaired mitophagy—contribute to the pathogenesis of conditions like osteoporosis, osteoarthritis, osteomyelitis, and delayed fracture healing. These alterations affect cellular processes including osteogenesis, inflammation, energy metabolism, and matrix synthesis.

Recent research highlights the potential of targeting mitochondria for treating various orthopedic disorders (Fig. 2, Fig. 3). For mitochondrial diseases, emerging therapies include dietary supplements, exercise, activation of mitochondrial biogenesis, antioxidant therapy, inhibition of mitochondrial fission and ROS accumulation and regulation of mitophagy [72]. While mitochondrial-targeted antioxidants such as mitoQ and SkQ1 have shown efficacy in preclinical models of bone loss and cartilage degeneration, clinical translation demands cautious evaluation [73,74]. Long-term antioxidant therapy may risk impairing physiological ROS-dependent signaling, which is critical for cellular adaptation and defense mechanisms. Existing human trials in non-orthopedic fields have generally demonstrated favorable safety profiles over months to a few years, but the potential consequences of prolonged use, particularly regarding immune competence and tissue regeneration, require further investigation in orthopedic populations. RNA-based therapeutic strategies, such as antisense oligonucleotides and RNAi drugs, are being explored for mitochondrial diseases, with potential delivery mechanisms including mitochondrion-targeted nanocarriers and endogenous mitochondrial RNA import pathways [75]. Another promising emerging therapeutic is Mdivi-1. Initially identified as an inhibitor of mitochondrial fission through blocking the activity of DRP1, Mdivi-1 has since been found to exert broader effects on mitochondrial function. It reduces mitochondrial reactive oxygen species (ROS) production, likely via reversible inhibition of Complex I of the respiratory chain. By modulating both mitochondrial structure and oxidative stress, Mdivi-1 is of growing interest for treating disorders involving mitochondrial dysfunction [76]. While some publications demonstrate an inhibition of mitophagy by Mdivi-1 [77], others state the exact opposite [78]. Although the exact effect on mitophagy is not yet fully understood, Mdivi-1 prevents bone loss in vivo [79]. The exact mechanism of action as well as its’ feasibility in the treatment of orthopedic conditions of Mdivi-1 is insufficiently understood and thus needs thorough further investigation. Additionally, defective mitochondria can be replaced by the emerging mitochondrial transplantation [80]. This involves isolating functional mitochondria from non-ischemic tissue and delivering them to the target area through direct injection or vascular infusion [81,82]. Preclinical studies and first clinical trials show promising results in the treatment of cardiac ischemia-reperfusion injury [80,83,84]. Although promising, mitochondrial transplantation faces significant translational challenges. Most current studies focus on cardiac and neurological tissues, with intraosseous or intraarticular applications yet to be systematically evaluated [85]. Practical hurdles include the method of mitochondrial isolation, delivery to target tissues, maintaining mitochondrial viability, and avoiding immune rejection [84]. Additionally, the long-term persistence and integration of transplanted mitochondria remain largely unknown, necessitating careful preclinical optimization before orthopedic clinical trials. A further treatment method is photobiomodulation (PBM). PBM involves the use of red to near-infrared light to stimulate beneficial cellular effects, primarily through interactions with mitochondria [86]. The primary photoacceptor is believed to be cytochrome *c* oxidase in the electron transport chain [87]. The proposed mechanisms include dissociation of inhibitory nitric oxide from cytochrome *c* oxidase and activation of light-sensitive ion channels [88]. This promotes tissue regeneration and reduces inflammation [89]. PBM has shown potential in treating neurodegenerative diseases, metabolic disorders, and nerve injuries by improving mitochondrial dynamics and bioenergetics [90,91]. It has also shown promising results in wound healing. So far, it has not been investigated in orthopedics. These advancements offer new potential in treating mitochondria-related disorders, although many therapies are still in early developmental stages.

### 2.1 Mitochondria in osteoarthritis

Mitochondrial dysfunction plays a significant role in osteoarthritis (OA), a common degenerative joint disease. Mitochondrial DNA polymorphisms and haplogroups influence OA prevalence, severity, and progression [92]. Mutations in mitochondrial DNA of chondrocytes lead to impaired OXPHOS, contributing to cartilage degeneration [93]. Mitochondrial dysfunction affects various cellular processes in OA, including increased oxidative stress, inflammation, and altered chondrocyte metabolism [94]. Targeting mitochondria represents a promising therapeutic approach for OA [95]. Potential interventions include antioxidant treatments, mitochondrial genome editing using CRISPR/Cas9 technology and mitochondrial transplantation [93]. However, challenges remain in developing effective and site-specific mitochondrial DNA editing techniques [93]. In posttraumatic OA, inhibiting mitochondrial oxidant production through intraarticular injection of amobarbital or N-acetylcysteine has shown promising results in preventing disease progression in animal models [96]. Other antioxidant treatment strategies include oral intake of natural products, such as curcumin or avocado-soya bean [97]. The reduced bioavailability and joint retention have led to the use of nanoparticles and hydrogels, with nanozymes showing potential to overcome physiological barriers [98]. Furthermore, Implantable and degradable antioxidant nanofibers have demonstrated effectiveness in OA treatment [99]. As research progresses, targeting mitochondrial metabolism may offer new strategies to impede or slow OA progression [94].

### 2.2 Mitochondria in osteoporosis

Recent research highlights the crucial role of mitochondrial dysfunction in the pathogenesis of osteoporosis and explores potential therapeutic options targeting mitochondria. It has been shown that mitochondrial membrane integrity, which is disrupted by Cyclophilin D, is involved in the pathogenesis of bone loss in osteoporosis, as Cyclophilin D knock-out mice exhibited enhanced resistance towards bone loss in an osteoporosis model [100]. Mitochondrial quality control mechanisms, including fusion, fission, biogenesis, and mitophagy, are essential for maintaining bone homeostasis [2]. Disruptions in these processes, along with mtDNA alterations and oxidative stress, contribute to osteoporosis development [12]. Sirtuins, particularly SIRT1, SIRT3, and SIRT6, have emerged as key regulators of mitochondrial quality control and bone metabolism, offering promising therapeutic targets [41]. Treatment strategies targeting mitochondria in osteoporosis focus on reducing oxidative stress. Mitochondria-specific antioxidants, such as MitoTEMPO, have shown potential in alleviating senescence of bone marrow mesenchymal stem cells and reducing bone loss in ovariectomized rats, a model of postmenopausal osteoporosis [12]. Mechanical stimulation and biophysical modulation of mitochondrial metabolism have also been found to promote bone anabolism and potentially combat osteoporosis [101].

Aging is a major contributor to mitochondrial dysfunction in bone, as evidenced by decreased mitochondrial DNA integrity, reduced OXPHOS efficiency, and diminished mitophagy in aging skeletal cells [102]. These impairments compromise the regenerative capacity of mesenchymal stem cells and osteoblasts, contributing to reduced bone formation and delayed fracture healing [103]. Age-related mitochondrial decline also increases susceptibility to oxidative stress and inflammation, further exacerbating bone fragility [102]. Understanding these aging-associated changes in mitochondrial function is critical for developing targeted interventions to preserve bone health in older populations.

It has been suggested, that mitochondrial transplantation can improve bone metabolism in osteoporosis [12]. Application of the mitochondria-derived MOTS-c peptide has demonstrated protective effects in an osteoporosis mouse model [104]. While further research is needed to ensure safety and efficacy, these mitochondria-targeted approaches present novel strategies for osteoporosis prevention and treatment.

### 2.3 Mitochondria in osteomyelitis

Current non-surgical therapies for osteomyelitis primarily focus on anti-infective approaches, including traditional antibiotics and novel agents targeting bacterial cell walls, membranes, and energy metabolism [105]. However, the rise of antibiotic resistance has prompted exploration of alternative treatments, such as bone-targeting compounds, antimicrobial surface modifications, and anti-virulence strategies [106]. Recent research highlights the crucial role of mitochondria in osteomyelitis. Studies reveal ultrastructural changes in mitochondria during osteomyelitis, including swelling, cristae depletion, and increased mitochondrial fission, resembling mitochondria in hypoxic tissues [71]. These alterations are linked to increased reactive oxygen species (ROS) production and cell death in infected bone cells [5]. Furthermore, research has demonstrated that increased mitochondrial fission is associated with osteogenic dysfunction [107]. Mitochondria are central to cellular energy production and proinflammatory responses, playing an essential role in defending infections [108]. However, excessive ROS production can lead to both mitochondrial and tissue damage. Pathogens exploit mitochondrial functions during infection, affecting OXPHOS and disrupting mitochondrial networks [108]. Mitochondria also regulate innate immune responses by hosting immune signaling modulators and promoting cellular metabolic reprogramming [109]. Understanding these mechanisms opens new perspectives for potential treatments targeting mitochondrial dynamics in osteomyelitis [71]. Despite their evident involvement in the disease, mitochondria are yet to be targeted in the treatment of osteomyelitis. Treatment strategies should aim to condemn mitochondrial ROS production and excessive mitochondrial fission and thereby promote osteogenic function and improve bone regeneration. This can be achieved by the administration of antioxidants or by the inhibition of fission. In this context, Mdivi-1—a mitochondrial fission inhibitor and ROS modulator—represents a promising therapeutic approach. Preclinical studies have shown its potential to suppress osteoclast formation and bone loss [79,110]. A complicating occurrence in chronic osteomyelitis is the formation of so-called biofilms. Biofilms are characterized by immense antibiotic resistance and immune evasion. Recent strategies to avoid the formation of biofilms include the use of antibacterial-coated implants [111]. Furthermore, hydrogel delivery of DNase I and liposomal vancomycin has shown promising results in a rat model of osteoporotic fracture-related infections [112]. However, coating strategies aim to eradicate infection without directly enhancing the regenerative capacity of bone tissue. Mdivi-1 may offer a potential adjuvant therapeutic to further improve the outcome of implant coatings in the treatment of osteomyelitis. Nevertheless, this remains speculative and requires further research to determine its feasibility and safety. This ought to be addressed in future studies.

### 2.4 Mitochondria in fracture healing

Mitochondrial treatments can aid in enhancing fracture healing through various mechanisms. Electromagnetic stimulation can increase mitochondrial function in osteogenic cells, promoting bone repair by activating OXPHOS [113]. Age-related delays in fracture healing are associated with reduced expression of mitochondrial genes, suggesting potential targets for intervention [114]. Inhibiting mitochondrial oxidant production after intra-articular fractures can prevent posttraumatic osteoarthritis, highlighting the importance of regulating mitochondrial metabolism in cartilage repair [96]. Additionally, mesenchymal stem cells overexpressing Lgr5 demonstrate enhanced osteogenic capacity and accelerated fracture healing, partly through regulation of mitochondrial dynamics [115]. Mitochondrial transfer to bone marrow mesenchymal stem cells improves their proliferation, migration, and osteogenic differentiation, promoting bone defect repair [116]. These studies collectively indicate that targeting mitochondrial function and related signaling pathways may offer novel therapeutic approaches for improving fracture healing and preventing associated complications. For example, the intravenous or intraosseous infusion of autologous mitochondria as well as the use of mitochondria-specific antioxidant agents may enhance fracture healing and better the outcome of complex fractures.

### 2.5 Mitochondria's role in wound healing

There is evidence that mitochondrial treatments potentially improve surgical outcomes in orthopedics. As mentioned above, recent research results indicate that targeting mitochondrial responses has potency to prevent posttraumatic osteoarthritis following intra-articular fractures by inhibiting oxidant production and maintaining chondrocyte metabolic function [96]. Mitochondria play a major role in the peri-implant microenvironment, influencing angiogenesis, macrophage immune responses, and bone formation during osseointegration [41]. It has been demonstrated that mitochondria are also involved in wound healing. The mitochondria-targeted antioxidant SkQ1 accelerated wound closure, enhanced epithelialization, and promoted granulation tissue formation in diabetic and aged mice [117]. Mitochondrial transplantation has also demonstrated therapeutic efficacy in human chronic pressure wounds [118]. PBM has shown promising results in the treatment of oral and diabetic wounds as it enhances cell migration, wound closure and antioxidant levels [89,119,120]. Thus, targeting mitochondria may improve the peri-implant microenvironment and post-surgical wound healing and thus could become an integral of future orthopedic surgeries. It is vital to thoroughly investigate this in future studies.

## 3 Conclusion

The role of mitochondria in orthopedics is pivotal for bone health, fracture healing, and surgical outcomes [[2], [3], [4]]. Mitochondria regulate the balance between osteogenesis and osteoclast activity, stem cell function, and immune responses in bone tissue [43,44]. Mitochondrial dysfunction is linked to musculoskeletal disorders such as osteoporosis, osteoarthritis, and osteomyelitis, underscoring the need for targeted interventions [12,71,94]. Recent advances have led to novel therapeutic strategies, including mitochondrial biogenesis stimulation, mitochondrial-targeted antioxidants, modulation of mitochondrial dynamics, mitochondrial transplantation, photobiomodulation and gene therapy [45,95,115]. These interventions show promise in preclinical studies for enhancing bone regeneration, reducing oxidative stress, and improving overall bone health. While still in early stages, these mitochondrial-targeted therapies require further research and clinical validation. Personalized treatments based on mitochondrial health and genetic background could enhance efficacy. Integrating these therapies into clinical practice could dramatically change the treatment of musculoskeletal diseases, offering new options for patients.

Among emerging strategies, mitochondrial transplantation has shown promise in restoring damaged mitochondrial networks. Although most studies have focused on cardiac and neurological tissues, its application via intraosseous or intraarticular delivery remains largely unexplored in orthopedics. Similarly, targeting mitochondrial fission with agents like DRP1 inhibitors has demonstrated potential in experimental models by improving bone regeneration and reducing osteoclastic bone loss. These approaches warrant further preclinical and clinical investigation to assess their feasibility, safety, and efficacy in musculoskeletal diseases.

In summary, targeting mitochondria represents a promising frontier in orthopedics, with the potential to address common bone disorders and improve patient outcomes. As research progresses, mitochondrial-based therapies may become integral to orthopedic care, promoting skeletal health and efficient healing processes.

## CRediT authorship contribution statement

**Daniel H. Mendelsohn:** Writing – review & editing, Writing – original draft, Visualization, Investigation, Formal analysis, Conceptualization. **Nike Walter:** Writing – review & editing, Conceptualization. **Wing-Hoi Cheung:** Writing – review & editing, Formal analysis. **Ronald Man Yeung Wong:** Writing – review & editing, Formal analysis. **Rebecca Schönmehl:** Writing – review & editing, Conceptualization. **Lina Winter:** Writing – review & editing, Conceptualization. **Thaqif El Khassawna:** Writing – review & editing, Formal analysis, Conceptualization. **Christian Heiss:** Writing – review & editing, Formal analysis. **Christoph Brochhausen:** Writing – review & editing, Visualization, Supervision, Investigation, Formal analysis, Conceptualization. **Markus Rupp:** Writing – review & editing, Visualization, Supervision, Investigation, Formal analysis, Conceptualization.

## Funding

This research received no external funding.

## Declaration of competing interest

The authors declare that they have no known competing financial interests or personal relationships that could have appeared to influence the work reported in this paper.

## Data Availability

No data was used for the research described in the article.
